# Species-Associated Differences in the Below-Ground Microbiomes of Wild and Domesticated *Setaria*

**DOI:** 10.3389/fpls.2018.01183

**Published:** 2018-08-21

**Authors:** Srinivasa Chaluvadi, Jeffrey L. Bennetzen

**Affiliations:** Department of Genetics, University of Georgia, Athens, GA, United States

**Keywords:** endophytes, Euryarchaeota, foxtail millet, metagenome, rhizosphere, root

## Abstract

The rhizosphere microbiome is known to play a crucial role in promoting plant growth, partly by countering soil-borne phytoparasites and by improving nutrient uptake. The abundance and composition of the rhizosphere and root-associated microbiota are influenced by several factors, including plant species and genotype. We hypothesize that crop domestication might influence the composition and diversity of plant-associated microbiomes. We tested the contribution of domestication to the bacterial and archaeal root and soil composition associated with six genotypes of domesticated *Setaria italica* and four genotypes of its wild ancestor, *S. viridis.* The bacterial microbiome in the rhizoplane and root endophyte compartments, and the archaea in the endophyte compartment, showed major composition differences. For instance, members of the Betaproteobacteria and Firmicutes were overrepresented in *S. italica* root samples compared to *S. viridis.* Metagenomic analysis of samples that contained both root surface-bound (rhizoplane) and inside-root (endophytic) bacteria defined two unique microbial communities only associated with *S. italica* roots and one only associated with *S. viridis* roots. Root endophytic bacteria were found in six discernible communities, of which four were primarily on *S. italica* and two primarily on *S. viridis*. Among archaea, Methanobacteria, and Methanomicrobia exhibited species-associated differences in the rhizosphere and root compartments, but most detected archaea were not classified more specifically than at the level of phylum. These results indicate a host genetic contribution to the microbial composition in *Setaria*, and suggest that domestication has selected for specific associations in the root and in the rhizosphere.

## Introduction

Plants, like all other organisms, are surrounded by massive numbers of microbes from a huge diversity of taxa (Thompson et al., 2017). The critical associations between plants and their microbes is the result of millions of years of co-evolution. Even with more than three decades of DNA-based diagnostic tools (Pace et al., 1985; Handelsman et al., 1998; Hugenholtz et al., 1998; Muyzer and Smalla, 1998; Lundberg et al., 2012; Peiffer et al., 2013; Edwards et al., 2015), only a small percentage of microbial ecotypes have been identified and only a tiny fraction of these studied to any great depth. For example, a recent study of the earth’s microbiome diversity indicated that >1/3 of metagenome reads from plant-associated microbial communities could not be mapped to existing rRNA databases (Thompson et al., 2017). This is not surprising, because sequence-based phylogenetic analysis of all of the ∼8000 prokaryotic genomes available in 2015 resulted in the identification of 79 bacterial phyla and 21 archaeal phyla (Parks et al., 2017), yet only four bacterial phyla have been identified by whole genome sequence analysis in ∼1000 phytobacterial genomes analyzed so far (Levy et al., 2018). This could be an accurate reflection of host ranges and/or an indication of the dearth of plant-associated studies conducted to date. Perhaps because plants do not have mobile immune cells or circulating antibodies, many microbes have established durable relationships within plant tissues. In some cases, these microbes provide useful services to the plant, such as for nitrogen fixation (Werner et al., 2015; Coskun et al., 2017) or mineral uptake (Bonfante and Genre, 2010; Smith and Smith, 2011) in the root. Many microbes are pathogenic to the plant, but these constitute only a tiny percentage of the total microbiome in plant::microbe interactions, while the great majority of plant-associated microbes provide either no services or unknown services.

Because soils are the most diverse source of microbes on the planet (Roesch et al., 2007; Trevors, 2010; Delmont et al., 2011), with as many as 10^4^ to 10^7^ unique bacterial cells per gram of soil, every root of a field-grown plant is bathed in as many as 10^11^ microbial cells per gram of root and more than 30,000 prokaryotic species (Berendsen et al., 2012; Bulgarelli et al., 2015). Numerous studies have shown that the microbial compositions of soils are dramatically altered by root growth, with the diversity significantly lowered and some microbes immensely increased in abundance in the rhizosphere (Reinhold-Hurek et al., 2015;Niu et al., 2017). These root-adjacent microbes can reach abundances of >10^9^ per gram of soil (Mendes et al., 2013) and are nurtured primarily by the exudates actively transported out of the plant root. These exudates, including organic acids, sugars, amino acids and many additional molecules, can amount to >50% of the energy/carbohydrate captured by the host plant through photosynthesis (Kuzyakov and Domanski, 2000; McNear, 2012). Hence, despite the fact that plants can be grown in sterile soils, these results suggest that soil microbes are of great importance for root and plant growth in real-world environments.

Many researchers believe that, like in the animal gut, a major role for microbial communities associated with the plant root is to physically displace pathogens and/or to generate antimicrobials against possible pathogenic microbes (Berendsen et al., 2012; Brader et al., 2017; Hacquard et al., 2017; Schlatter et al., 2017). This, of course, raises the issue of how plant::microbe interactions have co-evolved so that beneficial microbes are attracted and pathogenic microbes are discouraged. Part of the evolved plant component of this specificity is likely to involve specific signaling molecules, like the phenolics that attract and stimulate rhizobial (Cao et al., 2017) and mycorrhizal (Schmitz and Harrison, 2014) symbiosis. This signaling regimen creates a strong selection for pathogens, commensalists and mutualists (Hacquard et al., 2017) to also recognize these signals, similar to the case for the parasitic weed Striga that recognizes the same signal that attracts mycorrhizae (Lopez-Raez et al., 2017). Hence, an evolutionary arms race is expected between the host and pathogen in this process, as is also the case for specific recognition of pathogen effector molecules in plant disease resistance processes (Hacquard et al., 2017; Lopez-Raez et al., 2017).

Recent studies have shown that both host plant genetics and soil environment are major determinants of the microbial communities that abound in, on and/or near roots (Peiffer et al., 2013; Edwards et al., 2015; Goodrich et al., 2016). The plant genetic component is significant when different species are compared, but is also detected when different genotypes of the same plant species are investigated. This, then, opens the possibility that specific alleles of plant genes can be identified that determine which microbes are present in a plant::soil interaction. One dramatic variation in host plant genetics is associated with the suite of human-selected changes that are responsible for crop domestication (Wang et al., 2005; Doebley, 2006; Konishi et al., 2006; Cockram et al., 2007; Zhou et al., 2015). Most of the selected traits are for such agronomic properties as seed retention (shattering) and the day length dependence of flowering. However, there is some evidence that root traits might also be selected during domestication (Waines and Ehdaie, 2007). It makes sense that such factors as irrigation, monoculture or uniform plant spacing would put unique pressures on root development that might also affect root and rhizosphere microbial populations.

In the current study, we have chosen to use the *Setaria* model system, consisting of a developmentally plastic wild ancestor (*S. viridis*, green foxtail) and a domesticated descendant (*Setaria italica*, foxtail millet) (Brutnell et al., 2015). This study provides the first examination of possible effects of domestication upon the root and rhizosphere microbiomes in the genus *Setaria.* One previous study has investigated the rhizosphere microbiomes of several *S. italica* cultivars and in two locations (Jin et al., 2017). This investigation demonstrated that microhabitats and geographic location shape foxtail millet root microbial communities, but did not test for any possible role of host genetics on the root microbiome. The current study, in contrast, describes the different prokaryotic communities inside the root, on the root surface and in the surrounding rhizosphere. The results indicate that the domesticated and wild species differ in their microbes by plant genotype, but also show extensive variability in their microbiomes in replicated trials in the greenhouse and field. Six endophyte microbiome communities are identified in the greenhouse, of which four are primarily associated with *S. italica*.

## Materials and Methods

### Plant Growth Conditions and Microbial Sampling

Seeds from six accessions of *S. italica* (Yugu1, B100, 129-86, 84-96, 15-96, and 130-96) and four accessions of *S. viridis* [A10, SV9-2 (PI212625), PI230135, and 4-V (UMDEL)] were obtained from Prof. Katrien Devos. These seeds were collected from various field locations and USDA GRIN and multiplied in the University of Georgia Plant Biology Greenhouse by standard methods used for self-pollinated crops (Devos et al., 1998; Bennetzen et al., 2012; Mauro-Herrera et al., 2013; Schroder et al., 2017). The seeds were surface sterilized with 8% sodium hypochlorite (Bioworld, United States) for 10 min, followed by three rinses with sterile distilled water. Previous studies demonstrated that the disinfection of seed with 2-5% sodium hypochlorite solution eliminated any surface fungal or bacterial cells and spores (Sauer and Burroughs, 1986; Chun et al., 1997). Seed were germinated in circular-section pots (10″ in diameter and 10″ high) containing a 2:1 mixture of field soil collected from the University of Georgia farm in Bogart, GA and sand. The sand and soil mixture was steamed for 30 min. *Setaria* plants were grown in a greenhouse (with a 14 h photo-period and day-night temperatures of 26–20°C) and watered daily to approximately 70% soil water holding capacity. Each genotype was grown in three pots with each pot having three seedlings and the pots were randomly arranged. Neither pot location nor orientation were rotated on any specific schedule. Plants were grown for 30 days before the roots were harvested for metagenomic analyses.

Rhizosphere soil samples were taken from three individual plants (one per pot) of each genotype. Rhizosphere (Rh) was defined as the soil still attached to the roots after shaking the roots by hand, thus separating off soil not adhering tightly to the roots. Each root system with closely adhering soil was transferred to a 250 ml sterile flask containing 150 ml sterile distilled water. The bottles were shaken for 15 min. The rhizosphere soil fraction from the supernatant was precipitated after centrifugation of the extract for 30 min at 6,000 rpm and then stored at −80°C.

The washed roots were transferred to new 50 ml tubes and rinsed once again with sterile water and a portion of these roots was then stored at −80°C. This sample (from now on called REE) constitutes root surface-bound (ectophytic, i.e., rhizoplane) and inside-root (endophytic) bacteria. The other portion of the washed roots (∼2 g) was transferred to a 15 ml falcon tube with 5 ml phosphate buffer (pH 8.0), 50 μl lysozyme (100 mg/ml), 0.5 U Chitinase, 5 μl RNAseA (100 mg/ml), 2 g sterile 710–1180 micron beads, 2 g sterile 212–300 micron glass beads and shook at 250 rpm for 30 min @ 37°C. These roots were then washed gently with sterile water and stored at −80°C. This root sample, which is enriched for the root internal (endophytic) microbiome, is called REN.

### Amplification and Pyrosequencing

Each DNA sample was subjected to PCR with two sets of primers, each specific for amplification of rRNA sequences from either bacteria or archaea. For Illumina Miseq analyses, each metagenomic DNA sample used dual-indexed (Kozich et al., 2013) 16S rRNA primers to PCR amplify the V3–V4 region (Peiffer et al., 2013). The forward primer (5′–3′) had adapter sequence necessary for binding to the Illumina flow cell (underlined), i5 index sequence (x), binding sites for the Illumina sequencing primers (bold), two maximally degenerate bases (N) and conserved microbial primer 515F (caps) (aatgatacggcgaccaccgagatctacactctttccctXXXXXXXX**acacgacgctcttccgatct**NNGTGCCAGCMGCCGCGGTAA). Similarly, the reverse primers (5′–3′) had adapter sequences necessary for binding to the flow cell (underlined), i7 index sequence (X) to distinguish each sample, the Illumina sequencing primers (bold), and bacterial primer 806R (CAP) (caagcagaagacggcatacgagatXXXXXX**gtgactggagttcagacgtgtgctcttccgatct** GGACTACHVGGGTWTCTAAT). A complete list of unique index sequences is provided in **Supplementary Table S1**. The forward primer (5′–3′) to PCR amplify archaeal 16S rRNA gene sequence contained 454 Life Sciences primer B and conserved primer 109F sequences (ACKGCTCAGTAACACGT), while the reverse primer (GCCTTGCCAGCCCGCT XXXXXXXXXXX **ACCGCGGCKGCTGGC**) contained 454 Life Sciences primer A (underlined), a 11 base unique barcode (X) and the archaeal primer 529R (bold) for each sample. Further details regarding primers and barcodes used in bacterial and archaeal analysis are presented in **Supplementary Table S2**.

PCR reactions were performed in 50 μL with 100 ng of template DNA, 1 × Phusion High Fidelity Buffer (NEB, United States), 0.25 μM of each primer, 0.5 μM each dNTP, and 1U Phusion High-Fidelity Taq Polymerase (NEB, United States). Detailed information for primers and barcodes for each metagenomic sample is listed in **Supplementary Table S2**. PCR conditions were 98°C for 2 min; 26 cycles of 98°C for 10 s, 58°C for 10 s, 72°C for 30 s; with a final extension at 72°C for 10 min. Three replicate PCR reactions were performed for each sample with each primer pair. Replicate reactions were pooled and cleaned by using solid-phase reversible immobilization (SPRI) beads (Deangelis et al., 1995). DNA was quantified using a fluorometric kit (Quant-IT PicoGreen, Invitrogen). Equimolar quantities of each library (from 140 metagenomic sample libraries) were pooled. For Illumina Miseq, the 16S amplicon libraries were mixed up to 20% with Illumina-generated PhiX control libraries to artificially increase the genetic sequence diversity. The Illumina-MiSeq (PE250) run for the 16S amplicon library pool, and two runs of the Roche-454 sequencing of 16S archaeal amplicon libraries was performed at the Georgia Genomics and Bioinformatics Core (University of Georgia, Athens, GA, United States). Sequence data were submitted to Genbank under the Bioproject ID: PRJNA430270.

### Sequence Analysis and Tree Construction

Illumina Miseq data were analyzed using MOTHUR MiSeq SOP^1^, whereas Roche-454 data were analyzed with MOTHUR 454 SOP^2^. Briefly, sequence datasets were trimmed, clustered and classified in MOTHUR (Schloss et al., 2009) according to the following parameters: minimum length = 200 bp, maximum length = 500 bp, average quality score = 25, nucleotide mismatches in the primer sequence = 0, the maximum number of *N*s = 0. The trimmed sequences of bacterial and archaeal reads were further cleaned by removing chloroplast 16S rRNA reads and chimeric reads with remove.seq (using taxonomic classification) and UCHIME, respectively, in MOTHUR. Sequences were combined and aligned in MOTHUR using the full alignments of the rRNA small subunit sequences of the SILVA database as a template. The MOTHUR program was also used for preclustering, rarefaction analysis, distance calculations, clustering, and further analysis based on OTUs. A distance matrix was generated from the resulting sequences. Sequences were clustered into OTUs using the farthest-neighbor algorithm. Illumina 16S OTUs were further analyzed by retaining only abundant OTUs, defined as occurring >100 times in the entire dataset. For instance, a sample could have 101 reads in one sample, and it would be considered abundant, or it could have 21 reads in each of five samples, and would still be considered abundant. We rarified and normalized the reads by randomly subsampling 10,000 abundant reads from each sample. That is, differences in total read numbers between samples was not a factor in our analysis, so that all final values of OTU counts represent a percentage of an identical 10,000 reads from each sample.

### Data Analysis

Bacterial and archaeal data were analyzed separately using the phylogenetic framework provided within the suite of tools in the MOTHUR and Fast UniFrac program (Schloss et al., 2009; Hamady et al., 2010). Alpha diversity values for bacterial and archaeal sequence data were estimated using the Shannon diversity index and rarefaction curves. Principal Coordinate Analysis (PCoA) was performed using the Fast UniFrac metric (Hamady et al., 2010) and visualized as the clustering of samples from different treatments (e.g., *S. viridis* vs. *S. italica*) by using the JMP Pro. Both UniFrac and AMOVA were used to compare the microbial communities. These two methods test different hypotheses and can result in different *p*-values. Taxonomy-based classification of bacterial and archaeal 16S rRNA gene sequences was obtained using the RDP taxonomy and Bayesian classifier (Wang et al., 2007; Cole et al., 2014).

The color-coded Clustered Image Maps (CIMs) (“heat maps”) were generated using CIMminer (Scherf et al., 2000). CIMminer utilizes a hierarchical clustering algorithm based on the average-linkage method of Sokal (1958). The OTU tables were represented graphically by coloring each cell on the basis of the abundance of a particular OTU across different samples. A dendrogram is appended to the colored table to indicate the nature of the computed relationship among OTU abundances in the table. Species- and root compartment-specific metagenomic biomarkers were identified using the linear discriminant analysis (LDA) effect size (LefSe) method (Segata et al., 2011). LefSe determines the OTUs most likely to explain differences between classes by coupling standard tests for statistical significance with additional tests that factor in biological constancy and relevance. LefSe first uses the non-parametric factorial Kruskal–Wallis (KW) sum-rank test to detect features with significant differential abundance with respect to the sample of interest. Biological consistency is subsequently investigated using a set of pairwise tests among species/compartments using the (unpaired) Wilcoxon rank-sum test (Wilcoxon, 1945; Mann and Whitney, 1947). As a last step, LEfSe employs LDA (Fisher, 1936) to estimate the effect size of each differentially abundant OTU.

## Results

In our greenhouse experiments investigating *Setaria* growth in sterile and non-sterile soils, we have routinely observed seedling growth and root development differences between treatments and between *S. italica* and *S. viridis* responses (**Supplementary Figure S1**). In order to investigate the microbiomes of these *Setaria*, metagenome analyses were pursued.

In order to differentiate between very different below-ground plant::microbe interactions, all harvested root samples were used to generate three DNA sources. The first source was from soil washed from a root that had been pulled from the greenhouse pot (see section “Materials and Methods”). We call this sample the rhizosphere (Rh). The roots were separated into two aliquots, one treated with lysozyme and chitinase and the other untreated. The wash from the enzyme-treated roots did not produce any bacterial colonies on four different media that we tested. Thus, the root sample treatment should have removed most or all microbes on the root surface, and is thus called the root endophytic microbiome (REN). The untreated root sample is a mixture of root endophytes and ectophytes (attached to the root surface), so this sample is called REE.

We have generated Rh, REE, and REN samples from 18 plants of *S. italica* and 12 plants of *S. viridis*. Thus, a total of 90 sample types were collected from the greenhouse experiments. After DNA extraction, each sample is expected to contain both microbial genomic DNA and plant genomic DNA. Metagenomic analyses were then performed with either bacterial 16S ribosomal DNA primers or archaeal 16S ribosomal DNA primers. These primers were chosen because they do not extensively amplify host plant ribosomal DNA from either the organelles or the nucleus. These ribosomal DNA sequences were grouped into specific microbial operational taxonomic units (OTUs), which are equated with unique ecotypes when the degree of OTU similarity is >97%, which is a commonly used rule to define species by the metagenome research community (Mysara et al., 2017). Our first analyses were with sequences generated with 454 technology, but we then switched to Illumina data generation in order to get larger numbers of reads. The results shown are only for the Illumina data, but independent analyses of the two data sets yielded the same conclusions (data not shown), although these conclusions were less robust when only using the 454 data. OTU tables obtained from a total of 89 bacterial samples and 71 archaeal samples were used in the final analyses. The bacterial and archaeal OTU tables are shown in **Supplementary Tables S3**, **S4**.

Bacterial diversity was higher in the Rh than in the REE or REN compartments (**Supplementary Table S5**). We did not find a significant difference in the microbial diversity between *S. italica* and *S. viridis* samples (**Supplementary Figure S2A**). Rh samples have the most OTU types that were not found in other compartments (**Supplementary Figures S2B,C**). The root endophytic (REN) results have extensive overlap (that is, are a subset of) the Rh and REE samples. The differences between REE and REN results are the microbes tightly affixed to the root surface, an understudied component of the microbial soil::root interaction. To determine the microbes unique to the rhizoplane (“ectophytes”), we determined the abundant OTUs in both REE and REN compartments, and then found those that were unique to the REE. We found 25 abundant OTUs that are present only on the root surface (REE) of *S. italica* and 126 abundant OTUs that are present only in the REE compartment of *S. viridis* (**Supplementary Figures S2B,C**). We did not find any abundant genera in REN compartments of *S. italica* or *S. viridis* genotypes that were not also found in the REE samples. This was expected, given that OTUs in the REN samples (endophytes) should be a subset of the OTUs found in the REE (endophyte plus ectophyte) samples.

**FIGURE 1:**
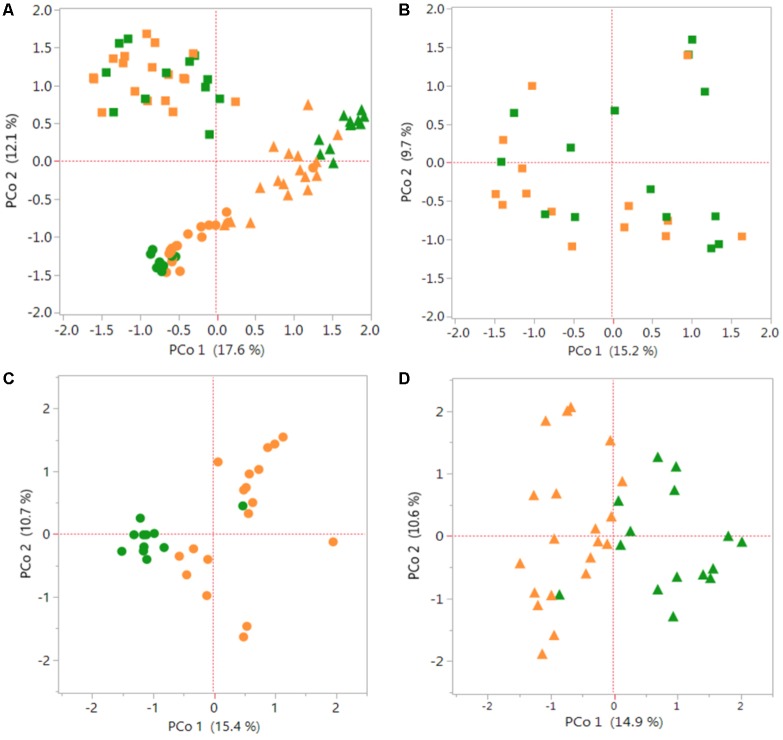
Species-dependent colonization of rhizosphere and root microbiomes in *Setaria* species. Bacterial OTUs of *Setaria italica* (orange) and *S. viridis* (green) were analyzed using Principal Coordinates Analysis (PCoA) plots generated from distance matrices for the Jaccard indices sets. The plot shows the first two principal axes. Rhizosphere (Rh) samples are shown as squares, root external plus root endophytic (REE) samples are shown as circles and root endophytic (REN) samples are shown as triangles. **(A)** Comparison of the three compartments across both species. **(B–D)** Figures are comparisons between species of the Rh, REE, and REN compartments individually. Non-parametric AMOVA to find significant differences between *S. italica* and *S. viridis* samples for each compartment produced *p*-values 0.056, 0.001, and 0.001 for Rh, REE, and REN samples, respectively.

The PCoA graphs in **Figure 1** shows a comparison of bacterial communities between the *S. italica* and *S. viridis* hosts in Rh, REE and REN compartments. Multiple genotypes of each species were used in the analysis, and three replicates of each genotype in most cases, so these results indicate differences that separate the domesticated plants from the wild plants. PCoA showed root compartment-specific and species-dependent separation. REE samples and REN samples showed significant species-dependent separation (**Figure 1A**). A separate analysis of only Rh samples (**Figure 1B**), only REE samples (**Figure 1C**) and only REN samples (**Figure 1D**) confirmed that both REE and REN samples showed significant species-dependent separation. A non-parametric analysis of variance (AMOVA) was performed to verify whether the differences in bacterial diversity between the groups (i.e., genotype, species, and root compartment) were different than within the groups. The result showed that there were statistically significant differences in bacterial diversity between species for REE and REN compartments (*p*-value ∼0.001). The analysis showed that the Rh compartment exhibits less significant host species-dependent microbial associations (*p*-value 0.046).

The presence and the abundances of different phyla and genera were assessed by taxonomic assignment of all the abundant sequences using the RDP classifier, and comparative analysis was conducted using MOTHUR tools. A total of 693 bacterial genera belonging to 23 phyla were recognized by RDP taxonomy. Out of these, 366 genera from 23 phyla were represented by more than 100 reads in the entire Illumina dataset. The top three phyla of Proteobacteria (76%), Actinobacteria (11%) and Firmicutes (9%) contributed 96% of the total bacterial diversity. Among Proteobacteria, the subphylum Alphaproteobacteria were the most abundant in the rhizosphere soil. In contrast, Deltaproteobacteria were the most abundant in the REE samples and Gammaproteobacteria were most abundant in the REN samples. These general abundance characteristics are very similar to those seen with many other root::soil communities (Bulgarelli et al., 2015).

**FIGURE 2:**
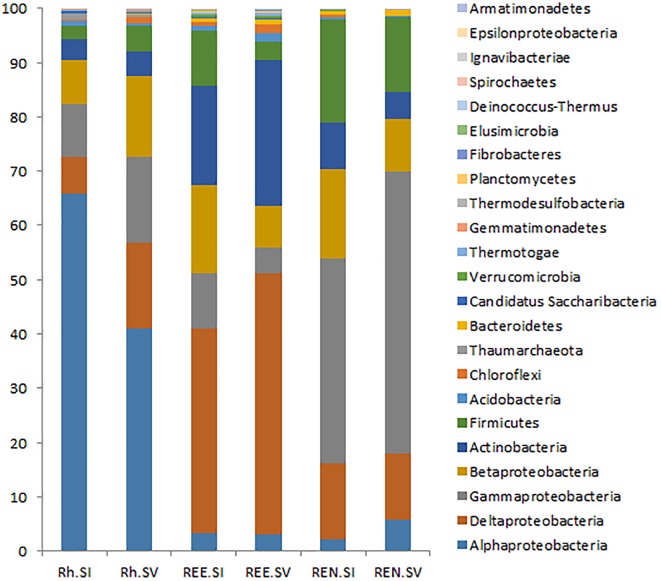
Relative abundance of bacterial phyla in the rhizosphere microbes (Rh), root external and endophytic microbes (REE) and root endophytic microbes (REN). The averages of each phylum for all *S. italica* samples (SI) and all *S. viridis* samples (SV) were shown as two bars for Rh, REE, and REN.

A comparative analysis of Rh samples from *S. italica* and *S. viridis* showed that Alphaproteobacteria were overrepresented in the *S. italica* rhizosphere, whereas Gammaproteobacteria, Deltaproteobacteria, and Firmicutes were overrepresented in the *S. viridis* rhizosphere soil (**Figure 2**). The REE fractions of *S. italica* roots were richer in Betaproteobacteria and Firmicutes than *S. viridis* roots. The REE fraction of *S. viridis* roots were found to be enriched with Deltaproteobacteria and Actinobacteria (**Figure 2**). The root endophyte compartment (REN) of *S. italica* was richer in Betaproteobacteria and Firmicutes compared to *S. viridis*. In contrast, the REN samples of *S. viridis* were enriched for Gammaproteobacteria (**Figure 2**). Sixteen genera (*Actinotalea, Algiphilus, Basilea, Caldivirga, Carnimonas, Ewingella, Hungatella, Mangrovibacter, Microvirga, Mitsuaria, Oxalicibacterium, Sphingobacterium, Succinivibrio, Taonella, Telmatocola, Xylophilus*), all abundantly present in rhizosphere soil, were completely absent in REE and REN compartments of both *S. italica* and *S. viridis*. Twelve genera (*Actinomyces, Desulfobaculum, Thiohalobacter, Polaromonas, Hamadaea, Edwardsiella, Peredibacter, Brevibacillus, Rivibacter, Thermocladium, Ochrobactrum, Mizugakiibacter*) that were abundant in REE compartments of both species were absent in the endophytic REN compartment.

**FIGURE 3:**
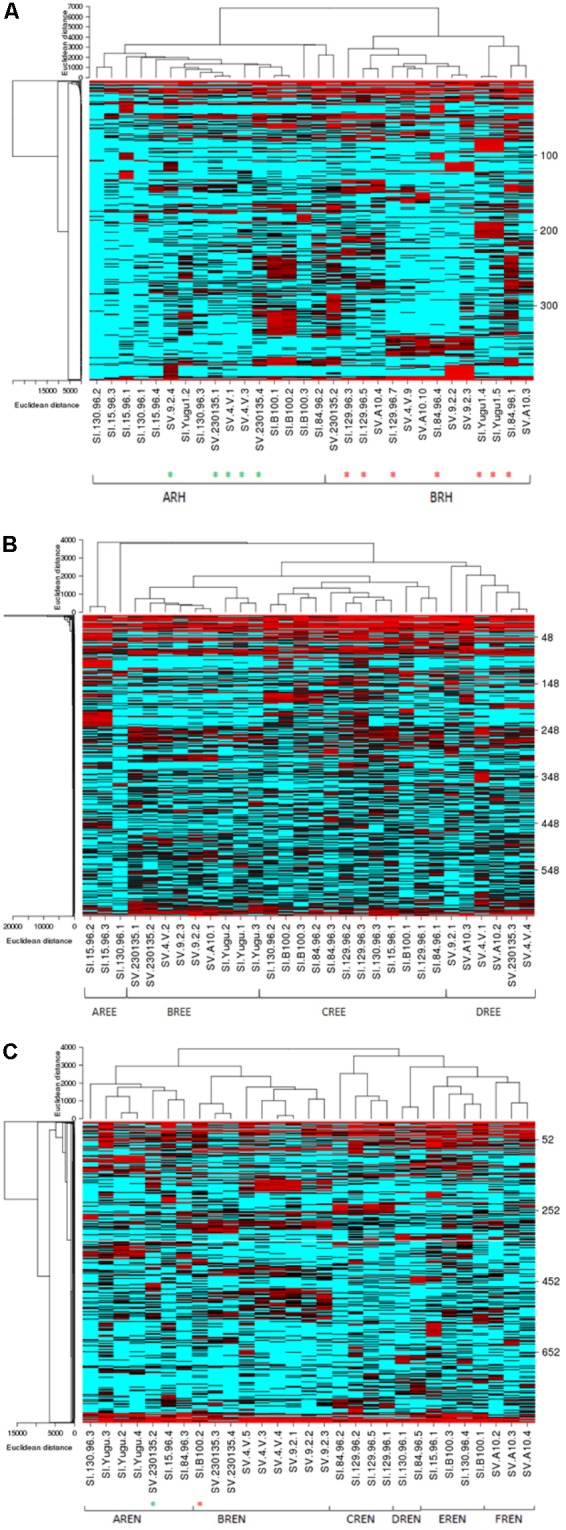
Species-dependent colonization of the rhizosphere (Rh) microbiome in *Setaria* species. Heat maps showing relative abundances of the most abundant bacterial OTUs in the Rh **(A)**, REE **(B)**, and REN **(C)** compartments (*Y*-axis) versus the *Setaria* genotypes (*X*-axis) used in the current study. Red indicates high abundance and blue indicates low abundance. The *Setaria* sample designations are on the bottom. The green stars at the bottom indicate *S. viridis* samples in an otherwise *S. italica* cluster and red stars indicate *S. viridis* samples in an otherwise *S. viridis* cluster. The *p*-values based on the Analysis of Molecular Variation (AMOVA) are 0.046^∗^, 0.001^∗^, and 0.001^∗^ for Rh, REN, and REE, respectively.

In order to characterize and depict the specific bacterial communities that are dominating these microbiomes, a heat map analysis is presented for the most abundant OTUs in the Rh, REE, and REN samples (**Figure 3**). We have also analyzed significant OTUs in each cluster by the LefSe method that predicts OTUs that are statistically different among biological samples. The significant OTUs in each heat map community are listed in the **Supplementary Table S6**.

The heat map analyses indicated two highly separable species-dependent microbiome communities in Rh samples (**Figure 3A**). The ARH community predominantly has *S. italica* samples with three *S. viridis* samples. The BRH community, which is found on most of the *S. viridis* samples, is also found on five *S. italica* samples. The OTUs coded as 1 (*Sphingomonas*), 41 (*Stakelama*), 67 (*Sphingomonas*), 126 (*Mesoaciditoga*), 128 (*Stakelama*), and 171 (*Stakelama*) in our analysis are significantly enriched in the ARH community compared to the BRH community, whereas 2 (*Lawsonia*), 3 (*Leclercia*), 5 (*Bhargavaea*), 6 (*Pseudomonas*) and 7 (*Massilia*) are most abundant in the BRH community.

The REE samples demonstrate four highly separable microbiome community types (**Figure 3B**). Each REE microbial community was completely species-dependent, with AREE and CREE communities only found with *S. italica* germplasm and BREE and DREE communities existing only with *S. viridis* roots. All of the replicates of four *S. italica* genotypes (Yugu1, B100, 84-96, and 129-96) were associated with the CREE community. All replicates of one *S. viridis* (A10) genotype were associated with the DREE community (**Figure 3B**). Some genotypes of *S. italica* and *S. viridis* associated with more than one type of microbial community. AREE has abundances of OTUs 27 (*Hathewaya*), 51 (*Rhizomicrobium*), 63 (*Clostridiaceae*), 111 (*Treponema*), and 142 (*Azospira*). BREE has a high proportion of OTUs 2 (*Lawsonia*), 220 (*Gimesia*), 776 (*Aquabacterium*), 1430 (*Aquabacterium*), and 1449 (*Pseudorhodoferax*), while CREE has high abundance of 46 (*Sporomusa*), 113 (*Succinivibrio*), 114 (*Anaerobacterium*), 159 (*Ideonella*) and 183 (*Anaerospora*). DREE contains high abundances of OTUs 9 (*Kitasatospora*), 35 (*Haliangium*), 492 (*Actinosynnema*), 614 (*Kallotenue*), and 786 (*Acidobacteria*).

The REN samples also have six distinct microbial communities, with AREN and CREN, DREN, and EREN within the roots of *S. italica* samples and BREN and FREN communities inside the roots of *S. viridis* samples (**Figure 3C**). All three replicates of Yugu1 had the AREN community and all the three replicates of SV4-2 and 9-2 have BREN communities, while A10 roots hosted the FREN microbial community. AREN exhibits over-representation of 6 (*Pseudomonas*), 10 (*Exiguobacterium*), 47 (*Pseudocitrobacter*), 62 (*Niastella*), and 72 (*Azorhizophilus*).BREN has overrepresentation of OTUs 1 (*Sphingomonas*), 3 (*Leclercia*), 73 (*Luteibacter*), 83 (*Achromobacter*), and 286 (*Siccibacter*), CREN has overrepresentation of 21 (*Tumebacillus*), 25 (*Janthinobacterium*), 37 (*Methylohalobius*), 318 (*Cedecea*), and 376 (*Massilia*), DREN has overrepresentation of 2 (*Lawsonia*), 4 (*Amycolatopsis*), 14 (*Byssovorax*), 17 (*Povalibacter*), 23 (*Mobilitalea*), EREN has significantly high proportion of OTUs 31 (*Serpens*), 86 (*Sulfuritalea*), 119 (*Acidobacteria*), 162 (*Serpens*), and 212 (*Leclercia*), while FREN has over abundance of OTUs 278 (*Enterobacter*), 674 (*Defluviitoga*), and 682 (*Puniceicoccus*).

**FIGURE 4:**
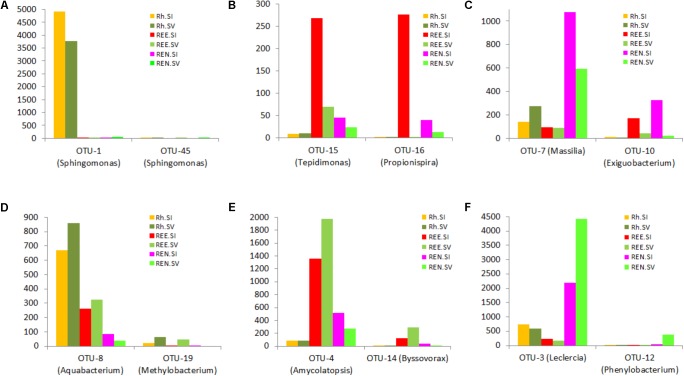
Species- and root compartment-specific significant OTUs were identified using the linear discriminant analysis (LDA) effect size (LefSe) method. A list of significant OTUs is presented in the Supplementary Data. This figure shows relative abundance of some exemplar biomarkers for *S. italica* Rh **(A)**, REE **(B)**, and REN **(C)** compartments and *S. viridis* Rh **(D)**, REE **(E)**, and REN **(F)**.

The OTU results were further analyzed to identify species-specific and compartment-specific biomarkers using the LefSe method. Six distintive (“biomarker”) OTUs were found for *S. italica* Rh, 14 for *S. viridis* Rh, 80 for *S. italica* REE, and 69 for *S. viridis* REE. In addition 51 biomarkers were found for the *S. italica* REN compartment, and 141 for the *S. viridis* REN compartment. The lists of significant OTUs are presented in **Supplementary Table S7**. The relative abundances of some of these exemplar biomarker OTUs are presented in **Figure 4**.

**FIGURE 5:**
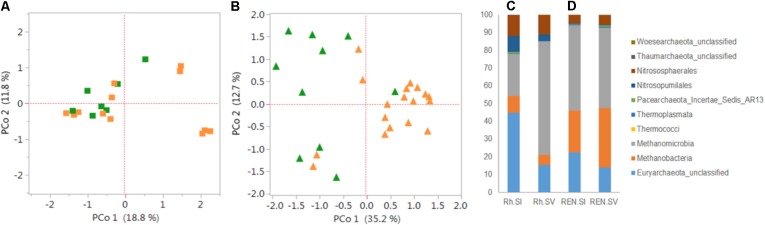
Diversity and composition of archaea in the roots and rhizospheres of *S. italica* and *S. viridis*. PCoA plots were based on 16S archaeal rRNA gene sequences for **(A)** rhizosphere microbiome (Rh) and **(B)** root endophytic microbiome (REN). The distance matrices were calculated from the Jaccard coefficients using Bray–Curtis indices. *P*-values calculated for the significance of the differences between *S. italica* and *S. viridis* in there results were based on unweighted UniFrac distances. The *p*-values were 0.12 and 0.016 for Rh and REN samples, respectively. The composition of archaeal classes for Rh samples **(C)** and REN samples **(D)** of *S. italica* and *S. viridis*.

The archaeal diversity was estimated in the Rh, REE and REN compartments of *S. italica* and *S. viridis* genotypes (**Supplementary Table S8**). *S. italica* roots have more archaea than *S. viridis* roots in all the compartments (**Supplementary Figure S3**). **Figure 5** presents the archaeal analyses on these same Rh and REN samples. PCoA demonstrated that the separation by domesticated versus wild *Setaria* genotypes in these experiments is more distinct in REN samples (**Figure 5B**) than in Rh or REE compartments (**Figure 5A** and **Supplementary Figure S4A**). Analysis of Rh and REN samples also indicated that the archaea predominantly belong to the phylum Euryarchaeota, with lower quantities of Thaumarchaeota. Euryarchaeota classes Methanomicrobia, Methanobacteria and an unknown class of Euryarchaeota constituted 80–90% of the Rh and REN archaea of both *Setaria* species (**Figure 5C** and **Supplementary Figure S4B**). Thaumarchaeota classes were represented by Nitrososphaerales and Nitrosopumilales.

## Discussion

Domestication of monocotyledonous crops resulted in changes in several traits, including seed size, seed yield, seed dispersal, determinate growth, loss of seed dormancy, and changes in photoperiod sensitivity (Doebley et al., 2006; Cockram et al., 2007; Zhou et al., 2015). Although these were all knowingly selected, there are no reports of selecting root architecture during domestication. However, there is evidence for an unknowing selection for less root biomass in domesticated wheat (Waines and Ehdaie, 2007), and decreases in the capacity for root plasticity under selection pressures in barley and foxtail millet (Grossman and Rice, 2012; Sebastian et al., 2016). *Setaria italica* is believed to have been domesticated from *S. viridis* in Northern China around 6000 BC (Bettinger et al., 2010), probably prior to rice domestication. It has been shown that some types of roots, such as the crown roots that originate from the shoot, show reduced sensitivity to water deficit in *S. italica* than in *S. viridis*, suggesting that this response has been influenced by human selection (Sebastian et al., 2016). Hence, there is a precedent for the idea that domestication can influence root traits.

Many factors are known and/or expected to influence the microbial communities associated with plant roots. The environment plays a major factor, especially soil chemical, structural and organic composition (Girvan et al., 2003; Fierer and Jackson, 2006). Cultivation history is also known to influence subsequent microbial communities, as simply exemplified by the abundance of rhizobiales and suppression of nitrifying microbial communities that are observed in a field that has recently supported a crop of legumes (Sugiyama et al., 2014; Paungfoo-Lonhienne et al., 2017). Soil composition can vary across very small scales, due to topographic issues like slope and altitude (Robertson et al., 1993; Kravchenko et al., 2005; Garcia-Palacios et al., 2012; Tamme et al., 2016). Hence, greenhouse experiments with controlled growth, soil and microbial composition conditions should offer substantial advantages for the generation of reproducible results. However, greenhouses are not as uniform as often assumed, particularly regarding edge issues of shading, distance to ventilation, local humidity variation and distances to exterior walls (and, thus, temperature variation), although these issues can be minimized by frequent pot translocation (Brien et al., 2013; Kutta and Hubbart, 2014). The greenhouse pot experiments in this study exhibited substantial environmental variation, as shown by differences in the replicates, but still yielded consistent signals regarding the differences between the microbiomes of domesticated versus wild *Setaria* prokaryotic ectophytes and endophytes. The higher abundance of Betaproteobacteria and Firmicutes in the REE and REN compartments of *S. italica* compared to *S. viridis* are clear examples. In addition, many significant species-specific OTUs belonging to these phyla were identified in the LefSe analyses.

Some of the different Rh, REE, and REN communities generated in this project show little to no overlap in their predominant community members, despite initiation of the community by identical microbial community inocula. Hence, the observed communities do not represent continuums, but rather the establishment of a defined community that show varying degrees of preference for a particular host genotype.

The microbial diversity in the REE and REN compartments showed substantial OTU overlap with the microbial communities in the Rh compartment, confirming the expectation that the REE and REN communities arising from a soil inoculation will be a subsample of Rh communities. Though seeds of all the genotypes in the current study were multiplied in the same potting mix in the same greenhouse, we cannot rule out the possibility that seed-transmitted microbes may have founded differences in the endophytic communities that were represented in the seedling root. However, previous work indicated that seed endophytic communities are distinctly different from rhizosphere or root microbial communities (Philippot et al., 2013; Leff et al., 2017).

There are several studies that have shown that the plant host genotype has a small but significant impact on the composition of an associated microbiome (Peiffer et al., 2013; Edwards et al., 2015). The host plant acquires its microbiome from the surrounding environments, but selects only a tiny subset of available species for associated growth. Soil type, biological history, water quality, water abundance and several other factors have important impacts on the microbes present as well. Establishing a novel microbiome takes time, so that greenhouse experiments on seedlings, especially on tiny seedlings like those of *S. viridis* and *S. italica*, are likely to show the smallest differentiation from the inoculum and the smallest host-specific effects. For instance, the host genotype-dependence of the microbiome was less apparent in a tiny annual such as Arabidopsis (Lundberg et al., 2012) than in a larger perennial plant, *Boechera stricta* (Wagner et al., 2016). Hence, we believe that the differences seen in our experiments would be more dramatic with larger and older plants than in these seedlings.

There are few studies that have directly assessed the possible effects of human agricultural intervention on the microbial communities in the roots. It was shown in wheat and maize that the root and rhizosphere bacterial communities are more diverse in land races than in the modern cultivars (Germida and Siciliano, 2001; Szoboszlay et al., 2015). However, it was shown in sunflower that there was less fungal diversity in the wild germplasm compared to domesticated genotypes (Leff et al., 2017). Leff et al. (2017) argued that the domestication of sunflowers may have decreased the prevalence of pathogens associated with the plants and may have increased the prevalence of symbionts. Our results suggest that the Betaproteobacteria, which include most of the nitrogen fixers in nature (Chen et al., 2003; Santi et al., 2013), are more abundant in the REE and REN compartments of the domesticated species, *S. italica*. Though we did not observe dramatic difference in the over-all microbial diversity between *S. italica* and *S. viridis*, we observed more microbial phylotypes specific to *S. viridis* than to *S. italica*. This could be a reflection of the broader adaptability of *S. viridis*, one of the two or three most widely distributed weeds on the planet, than seen for *S. italica*, which was domesticated and primarily grown in only one region of China. All crop domestication and improvement are expected to be associated with the narrowing of germplasm, so this phenomenon would also predict less diversity of all kinds (including the associated microbiome) in an elite crop.

Previous studies have postulated that ancient cultivars and their wild relatives were generally exposed to more marginal soils before the invention of synthetic fertilizer–driven agricultural production, and their gene pools might have a different adaptive capacity to engage in novel microbial associations with rhizosphere microbes compared with the gene pools of present-day cultivars (Wissuwa et al., 2009; Bulgarelli et al., 2015). These observations suggest that identification of the alleles in *S. viridis* that promote root::soil microbe diversity might also contribute to improved adaptation of the crop foxtail millet to diverse environments. Mapping and introgression of the *Setaria* genes responsible for these root::soil microbe differences will be needed to test this hypothesis.

## Conclusion

This study demonstrates that host genetics, including the outcomes of crop domestication, can play an important role in selecting the prokaryotes present in the plant::soil interaction network. The communities established in the rhizosphere, root surface and endophytic space are very different, but are all affected by both host genetics and the plant microenvironment. Now that these differences have been observed, future field experiments and experiments with controlled innocula can be used to determine the contributions of individual microbial species to the community and to plant performance.

## Author Contributions

JB designed the experiments, analyzed the data, and wrote the manuscript. SC designed and conducted the experiments, analyzed the data, and wrote the manuscript.

## Conflict of Interest Statement

The authors declare that the research was conducted in the absence of any commercial or financial relationships that could be construed as a potential conflict of interest.

## Footnotes

1 https://www.mothur.org/wiki/MiSeq_SOP

2 https://www.mothur.org/wiki/454_SOP
