# Supplementary material for: Species-Associated Differences in the Below-Ground Microbiomes of Wild and Domesticated Setaria
Source: Front Plant Sci. 2018 Aug 21;9:1183. doi: 10.3389/fpls.2018.01183 (PMC6111228; doi:10.3389/fpls.2018.01183)
Supplement: Supplementary file 9 [file Presentation_1.pptx]

## Slide 1
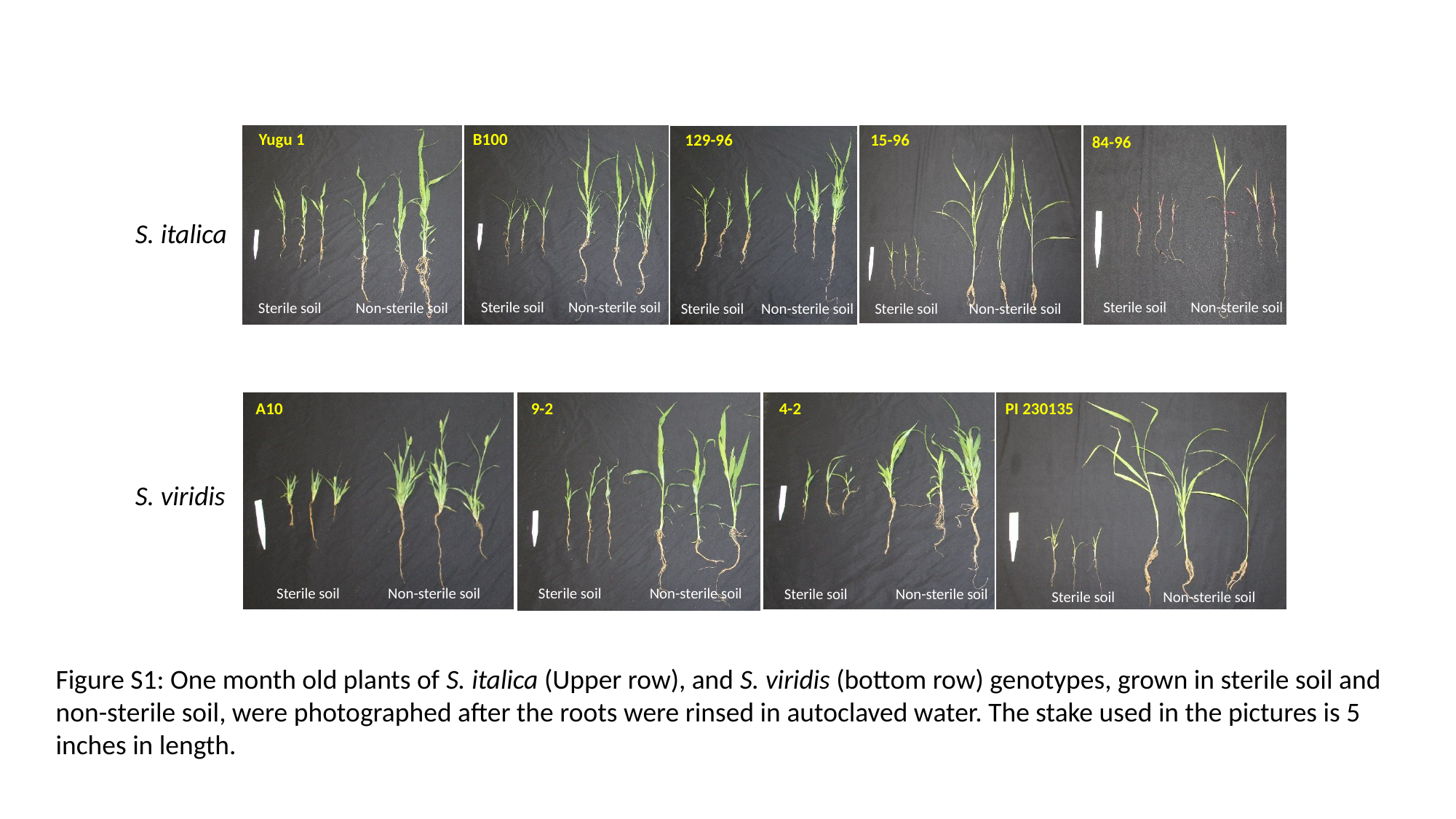

Yugu 1
B100
129-96
15-96
84-96
S. italica
S. viridis
Sterile soil Non-sterile soil
Sterile soil Non-sterile soil
Sterile soil Non-sterile soil
Sterile soil Non-sterile soil
Sterile soil Non-sterile soil
9-2
4-2
PI 230135
A10
Sterile soil Non-sterile soil
Sterile soil Non-sterile soil
Sterile soil Non-sterile soil
Sterile soil Non-sterile soil
Figure S1: One month old plants of S. italica (Upper row), and S. viridis (bottom row) genotypes, grown in sterile soil and non-sterile soil, were photographed after the roots were rinsed in autoclaved water. The stake used in the pictures is 5 inches in length.

## Slide 2
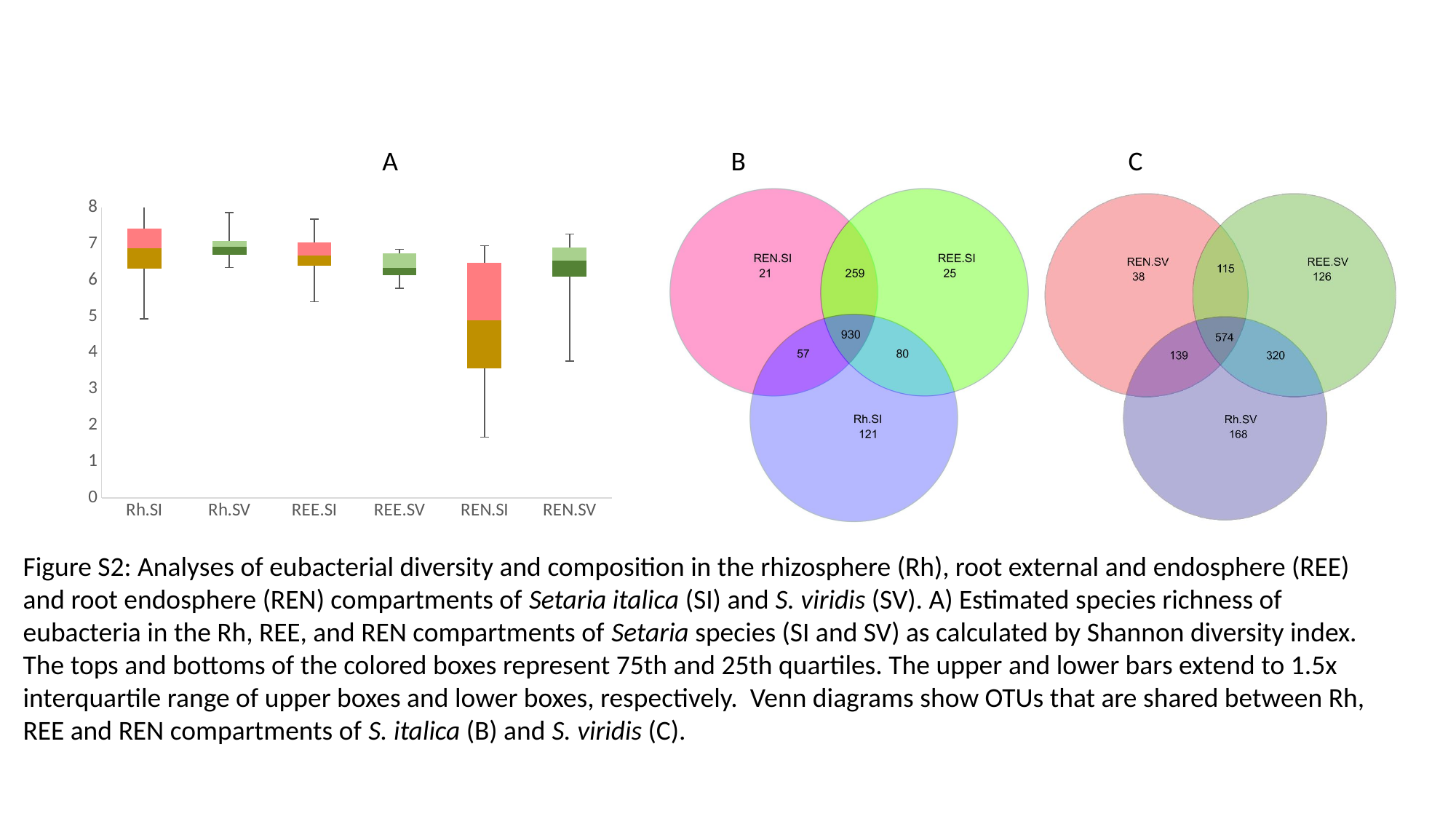

A B C
### Chart
| Category | Series 1 | Series 2 | Series 3 | Series 4 |
|---|---|---|---|---|
| Rh.SI | 4.930134999999999 | 1.38755125 | 0.569520249999999 | 0.526345000000001 |
| Rh.SV | 6.351831999999999 | 0.356865 | 0.213508583333333 | 0.150083416666667 |
| REE.SI | 5.409073 | 0.99887025 | 0.267400749999999 | 0.3636335 |
| REE.SV | 5.779978 | 0.350567 | 0.213685083333334 | 0.404235916666666 |
| REN.SI | 1.669478 | 1.9006205 | 1.3244705 | 1.592374 |
| REN.SV | 3.770262 | 2.325837250000001 | 0.43382475 | 0.361864 |
Figure S2: Analyses of eubacterial diversity and composition in the rhizosphere (Rh), root external and endosphere (REE) and root endosphere (REN) compartments of Setaria italica (SI) and S. viridis (SV). A) Estimated species richness of eubacteria in the Rh, REE, and REN compartments of Setaria species (SI and SV) as calculated by Shannon diversity index. The tops and bottoms of the colored boxes represent 75th and 25th quartiles. The upper and lower bars extend to 1.5x interquartile range of upper boxes and lower boxes, respectively. Venn diagrams show OTUs that are shared between Rh, REE and REN compartments of S. italica (B) and S. viridis (C).

## Slide 3
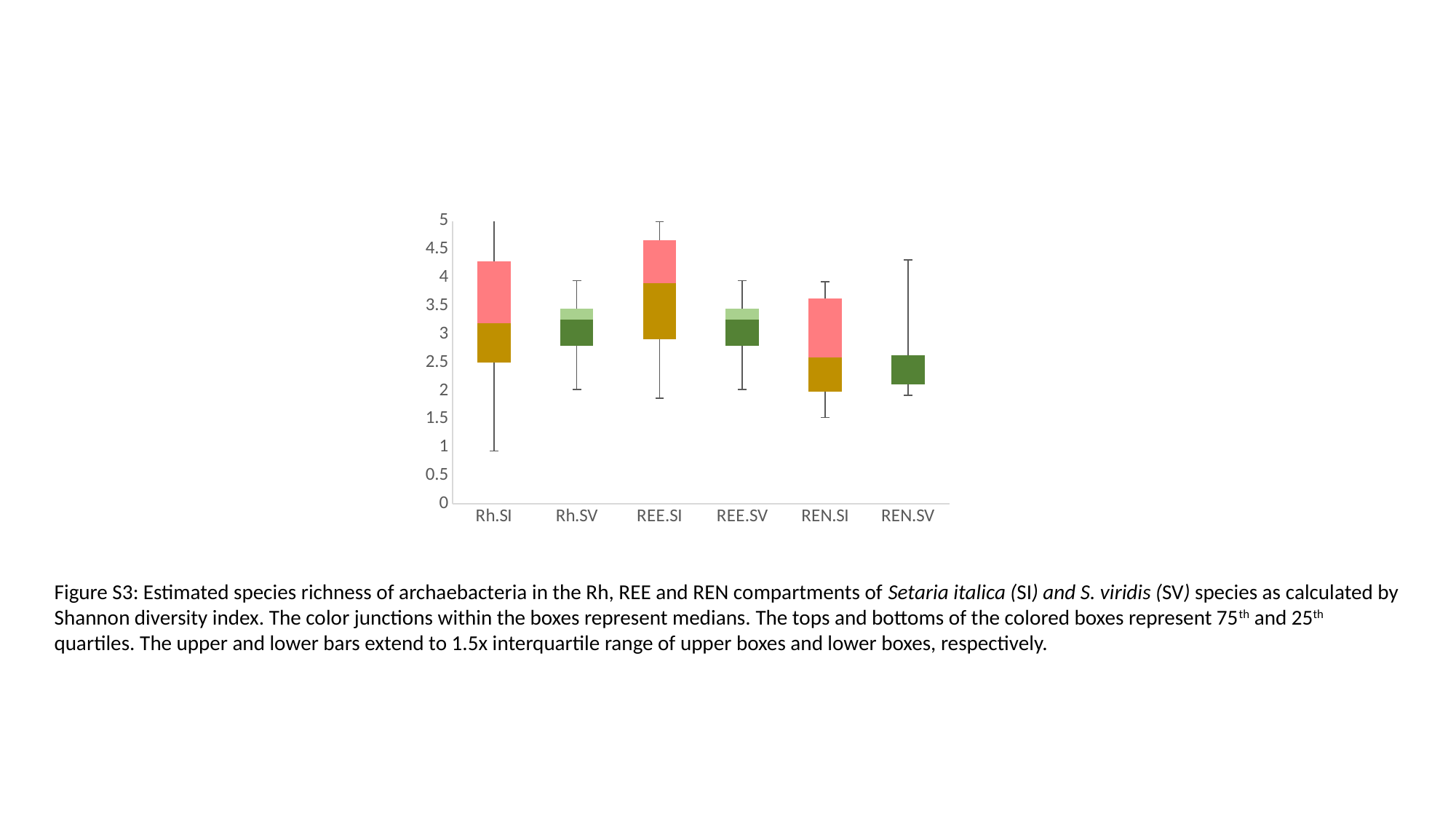

### Chart
| Category | | | | |
|---|---|---|---|---|
| Rh.SI | 0.933857 | 1.560308 | 0.695299 | 1.098511 |
| Rh.SV | 2.024659 | 0.77622975 | 0.45290625 | 0.1923135 |
| REE.SI | 1.867276 | 1.04732575 | 0.98628725 | 0.761005 |
| REE.SV | 2.024659 | 0.77622975 | 0.45290625 | 0.1923135 |
| REN.SI | 1.529145 | 0.4488465 | 0.6088215 | 1.04821275 |
| REN.SV | 1.918403 | 0.196583 | 0.5120825 | 0.0 |Figure S3: Estimated species richness of archaebacteria in the Rh, REE and REN compartments of Setaria italica (SI) and S. viridis (SV) species as calculated by Shannon diversity index. The color junctions within the boxes represent medians. The tops and bottoms of the colored boxes represent 75th and 25th quartiles. The upper and lower bars extend to 1.5x interquartile range of upper boxes and lower boxes, respectively.

## Slide 4
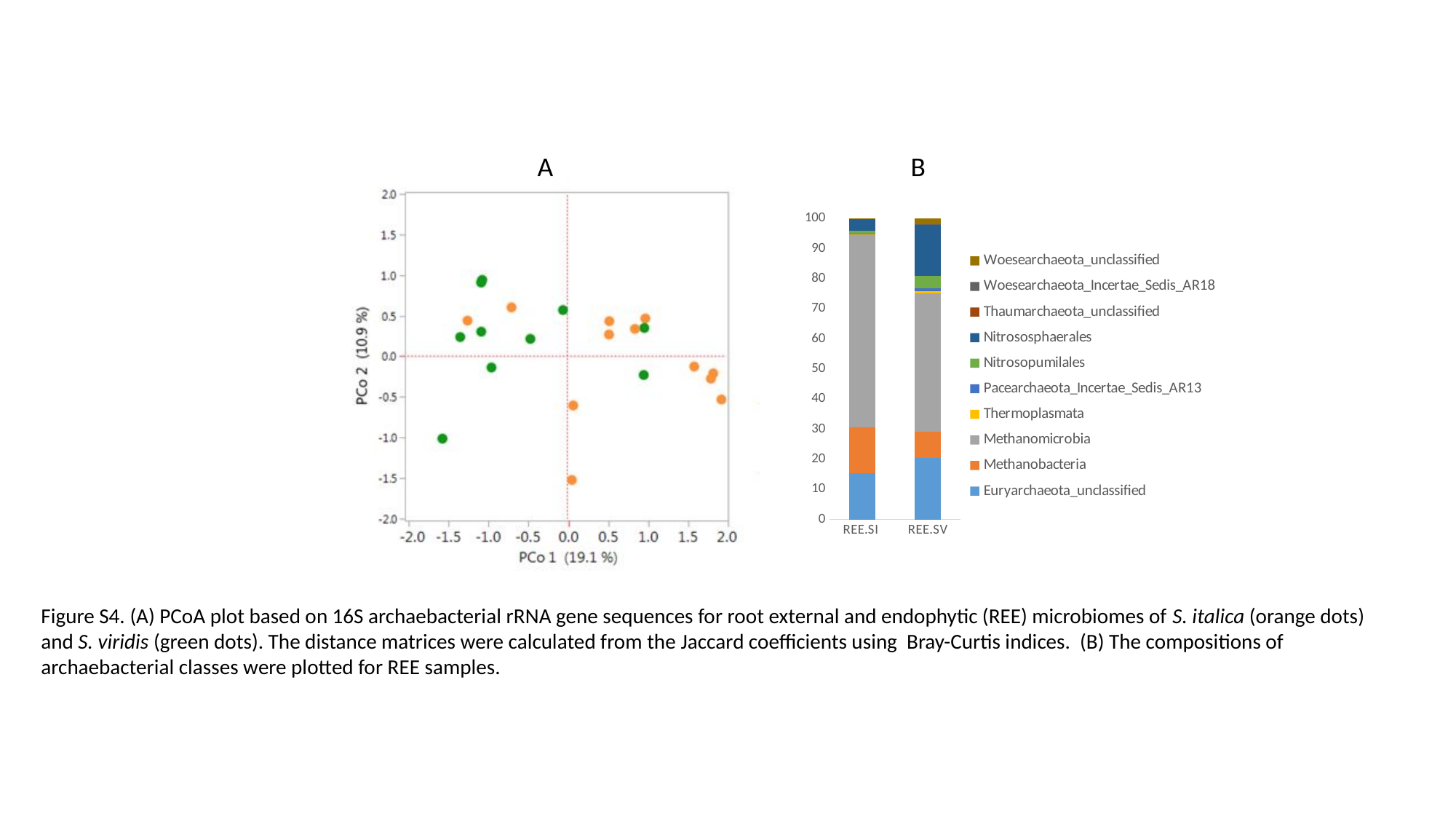

A B
### Chart
| Category | Euryarchaeota_unclassified | Methanobacteria | Methanomicrobia | Thermoplasmata | Pacearchaeota_Incertae_Sedis_AR13 | Nitrosopumilales | Nitrososphaerales | Thaumarchaeota_unclassified | Woesearchaeota_Incertae_Sedis_AR18 | Woesearchaeota_unclassified |
|---|---|---|---|---|---|---|---|---|---|---|
| REE.SI | 15.32531606537156 | 15.20197348134442 | 63.82978723404251 | 0.431699044094974 | 0.0616712920135676 | 0.940487203206907 | 4.085723095898858 | 0.0154178230033919 | 0.0 | 0.107924761023743 |
| REE.SV | 20.46470062555853 | 8.579088471849866 | 46.02323503127793 | 0.774501042597557 | 0.953232052427763 | 4.110813226094726 | 17.15817694369973 | 0.0595770032767352 | 0.0297885016383676 | 1.846887101578791 |Figure S4. (A) PCoA plot based on 16S archaebacterial rRNA gene sequences for root external and endophytic (REE) microbiomes of S. italica (orange dots) and S. viridis (green dots). The distance matrices were calculated from the Jaccard coefficients using Bray-Curtis indices. (B) The compositions of archaebacterial classes were plotted for REE samples.
